# Clinical characteristics and outcomes for pregnant women diagnosed with COVID-19 disease at the University of Benin Teaching Hospital, Benin City, Nigeria

**DOI:** 10.11604/pamj.2021.39.134.27627

**Published:** 2021-06-16

**Authors:** James Osaikhuwuomwan, Michael Ezeanochie, Charles Uwagboe, Kingsley Ndukwu, Sofiat Yusuf, Adedapo Ande

**Affiliations:** 1 Department of Obstetrics and Gynaecology, University of Benin Teaching Hospital, Benin City, Edo State, Nigeria,; 2 Materno-Foetal Unit, University of Benin Teaching Hospital, Benin City, Edo State, Nigeria

**Keywords:** COVID-19, coronavirus, pregnancy, Nigeria

## Abstract

**Introduction:**

the novel coronavirus disease (COVID-19) pandemic has challenged health systems around the world. This study was designed to describe the socio-demographic characteristics of pregnant women with COVID-19 infection, the common clinical features at presentation and the pregnancy outcome at the University of Benin Teaching Hospital, Edo State, Nigeria.

**Methods:**

a cross-sectional analytical study of all confirmed cases of COVID-19 infection from April to September 2020.

**Results:**

out of 69 suspected cases that were tested, 19 (28.4%) were confirmed with COVID-19 infection. The common presenting complaints were fever (68.4 %), cough (57.9 %), sore throat (31.6%), malaise (42.1%), loss of taste (26.3%), anosmia (21.1%), and difficulty with breathing (10.6%). In terms of treatment outcome, 57.9% delivered while 36.8% recovered with pregnancy on-going, and 1 (5.3%) maternal death. Of the 11 women who delivered, 45.4% had vaginal deliveries and 54.6 % had Caesarean section. The mean birth weight was 3.1kg and most of the neonates (81.8%) had normal Apgar scores at birth. There was 1 perinatal death from prematurity, birth asphyxia, and intrauterine growth restriction. The commonest diagnosed co-morbidity of pregnancy was preeclampsia and it was significantly associated with severe COVID-19 disease requiring oxygen supplementation (P = 0.028).

**Conclusion:**

the clinical symptoms of COVID-19 in pregnancy are similar to those described in the non-pregnant population. It did not seem to worsen the maternal or foetal pregnancy outcome. The occurrence of preeclampsia is significantly associated with severe COVID-19 infection requiring respiratory support.

## Introduction

The coronavirus disease (COVID-19) pandemic has challenged health systems around the world. The disease is caused by the Severe Acute Respiratory Syndrome Coronavirus 2 (SARS-CoV-2) infection [1]. It is a novel respiratory disease that is highly infectious and may lead to multiple organ failure and death [1]. The emergence and rapid spread of the new coronavirus across the world poses severe threat to global public health [1,2]. Symptoms that may be associated with COVID-19 disease include fever, dry cough, sore throat malaise, loss of sense of taste and smell, shortness of breath and respiratory distress [2,3]. Measures such as physical distancing, movement restrictions and “lock downs” were instituted by various countries to curb the rapid spread of the disease [4,5]. These interventions had implications for routine healthcare services which suffered disruptions as health systems struggled to cope with the effects of the pandemic. Furthermore, while data from previous coronavirus outbreaks documented pregnancy as a risk factor for worse outcomes, early reports from COVID-19 infection in pregnancy appear to be different [6]. While studies have associated COVID-19 infection in pregnancy with increased risk of miscarriage, foetal growth restriction, and preterm birth [7-9], other published results have not observed any significant effects of the disease on obstetric and neonatal outcome [10,11].

A recent systematic review with meta-analysis noted that while previous coronavirus outbreaks (Severe Acute Respiratory Syndrome (SARS) and Middle East Respiratory Syndrome (MERS)) have associated infections during pregnancy with more serious illness, the maternal morbidity for COVID-19 infection during pregnancy appears like that of non-pregnant women of reproductive age [10,11]. It recommends the need for more evidence to sufficiently address the relationship between COVID-19, pregnancy, and the effects on maternal and neonatal outcomes. The maternity unit of the University of Benin Teaching Hospital, Benin City is a major referral obstetric unit in Southern Nigeria. It continued to render obstetric services during the pandemic and was also designated as a centre to manage patients with COVID-19 infection during pregnancy. This study was conducted to document our experience with the management of pregnant women with COVID-19 infection during pregnancy. It explores the socio-demographic characteristics of the women, clinical features at presentation, the associated comorbidities and maternal-foetal pregnancy outcomes. This will add to the literature; existing-cum-emerging scientific information on this novel coronavirus infection and contribute to improving the quality of care provided to pregnant women affected with the infection.

## Methods

**Study setting:** the University of Benin Teaching Hospital is a major referral tertiary hospital in Southern Nigeria and is one of the designated hospitals for the management of COVID-19 disease in Edo State, Nigeria. The Department of Obstetrics records about 2,600 deliveries annually and continued to provide maternal healthcare services to pregnant women during the pandemic. Services were provided in line with the periodically updated guidelines and protocols from the Nigerian Centre for Disease Control. Patient selection for testing, sample collection, processing, and laboratory testing also followed guidance from Nigeria Centre for Disease Control (NCDC) on COVID-19 [5]. All suspected patients had laboratory test for COVID-19 infection via nasopharyngeal or throat swab test for Severe Acute Respiratory Syndrome Coronavirus 2 (SARS-CoV-2) by using quantitative reverse transcription polymerase chain reaction (qPT-PCR) [5]. Positive cases were either admitted to the isolation ward for inpatient care or managed from home on supervised self-isolation based on the NCDC guidelines. Additionally, suspected or confirmed cases in labour were managed in specially designated delivery suites specified for that purpose within the labour ward complex. Ethical approval for this study was obtained from the UBTH Health and Research Ethics Committee.

**Study design and patients:** this was a descriptive observational study of all suspected cases of COVID-19 infection that tested positive during the study period from April to September 2020. We retrospectively reviewed the clinical features and outcomes of all pregnant women with COVID-19 infection. Obstetric cases flagged as suspected COVID-19 cases were tested during the study period. The cases that were confirmed COVID-19 positive constitute the study population.

**Data analysis:** clinical information for the COVID-19 positive patients were collated for analysis. The variables of interest were their socio-demographic and clinical characteristics, laboratory tests results and outcome of treatment (recovery and discharge or death). For those who had delivered, information on pregnancy and neonatal outcomes were retrieved. Data was obtained from the case records, labour ward records, labour ward theatre records and the postnatal ward. The data was analysed using the SPSS software (v.20.0, IBM SPSS). The results are presented as frequency tables with percentages and proportions. Student´s test was performed to compare continuous variables while Chi-square test and Fisher´s exact test were used to compare categorical variables. Statistical significance was set at p value < 0.05.

## Results

Overall, there were 67 obstetric patients with suspected COVID-19 symptoms for which nasopharyngeal samples for RTPCR for SARS-CoV-2 nucleic acid were taken. Out of these number, 19 (28.4%) were confirmed with COVID-19 infection. In terms of treatment outcome, 11 women (57.9%) delivered while 7 (36.8%) recovered and were discharged (pregnancy on-going), and 1 (5.3%) maternal death was recorded. Two women (10.5%) had preterm delivery. In Table 1, the clinical profile of the study population is presented. The mean age of the positive cases was 31.4 years (ranged from 22-39) and most (52.6%) were within 31-35 years age group. Most (63.2%) of the women were multipara (Para 1-4), 78.9% were educated beyond the secondary school level (tertiary education), and 47.4% were skilled professionals. Infection with COVID-19 was mostly diagnosed during the antenatal period (78.9%).

**Table 1:** clinical profile of COVID-19 positive women

Variable	Number (%)
Age: mean ± SD (years)	31.4 ± 4.1
Age interval (years)	
21-25	1 (5.3)
26-30	5 (26.3)
31-35	10 (52.6)
36-40	3 (15.8)
Parity:	
Para 0	5 (26.3)
Para 1-4	12 (63.2)
Para ≥ 5	2 (10.5)
Education	
Primary	1 (5.3)
Secondary post-secondary (tertiary)	3 (15.8) 15 (78.9)
Period at diagnosis	
Antenatal	15 (78.9)
Intrapartum	2 (10.5)
Postpartum	2 (10.5)
Gestational age (weeks)	
Mean (SD)	30.4 (7.7)
Range	12-40
Trimester of pregnancy (at diagnosis)	
First	1 (5.3)
Second	5 (26.3)
Third	13 (68.4)

The clinical manifestation of the women is described in Table 2. The common presenting complaints were fever 13/19 (68.4 %) and cough 11/19 (57.9 %). Other clinical symptoms observed were sore throat 31.6% (6/19), malaise 42.1% (8/19), loss of taste, 26.3% (5/19), anosmia 21.1% (4/19), difficulty with breathing 10.55%(2/19). The most common diagnosed co-existing morbidity was hypertensive disorder in pregnancy 26.3% (5/19) while 13 (68.4%) were in the third trimester of pregnancy. In Table 3, the maternal and foetal treatment outcome is presented. Five (26.3%) women received oxygen supplementation while 1(5.3%) required intensive care unit (ICU) admission. Out of the 11 women who delivered, 5 (45.4%) had vaginal delivery and 6 (54.6 %) Caesarean section; 4 (36.4%) emergency and 2 (18.2 %) elective Caesarean sections. The mean birth weight was 3.1kg (ranged from 1.2-4.2) and most of the neonates 9 (81.8%) had normal Apgar scores at birth. Two neonates (18.2%) were admitted into the neonatal unit for birth asphyxia and 1 suffered early neonatal death within 24 hours of birth, In Table 4, the relationship between disease severity (requirement for respiratory support) and presence of co-morbidities in pregnancy was compared. There was a statistically significant difference in the occurrence of severe COVID-19 infection among women who had comorbidities in pregnancy (P = 0.028).

**Table 2:** common clinical manifestations

Variable	N (%)
Fever	13 (68.4)
Cough	11 (57.9)
Sore throat	6 (31.6)
Malaise	8 (42.1)
Anosmia	4 (21.1)
Loss of smell	5 (26.3)
Difficult breathing	3 (15.8)
Asymptomatic	2 (10.5)
Associated co-morbidities*:	
Preeclampsia/eclampsia	5 (26.3)
Retroviral disease	1 (5.3)
Acute pyelonephritis	1 (5.3)
Preterm premature rupture of membranes (PROM)	2 (10.5)
Peuperal sepsis	1 (5.3)

* Some patients had multiple co-morbidities

**Table 3:** maternal and foetal treatment outcome

Variable	Number (%)
Treatment	
Inpatient care	10 (52.6)
Outpatient care	9 (47.4)
Used oxygen	5 (26.3)
ICU admission	1 (5.3)
Obstetric outcome	
Full recovery and delivered	11 (57.9)
Full recovery ongoing pregnancy	7 (36.8)
Maternal death	1 (5.3)
Mode of delivery	
Vaginal delivery	5 (26.3)
Caesarean section	6 (31.6)
Birth weight (kg):	
Mean ± SD	3.1 ± 0.8
Range	1.2-4.2
Birth asphyxia	
None	9 (81.8)
Mild	1 (9.1)
Moderate	1 (9.1)
Severe	0 (0)
Admission into neonatal unit	2 (10.5)
COVID-19 positive neonate	0 (0)
Early neonatal death	1 (5.3)

**Table 4:** association between disease severity and detected comorbidities of pregnancy

Variable	Severe disease used oxygen n = 5	Mild disease no oxygen n = 14	Test statistics	P-value
Obstetric co-morbidity				
None	1	11		
Preeclampsia/eclampsia	3	2		
Acute pyelonephritis	0	1	10.835	0.028
Puerperal sepsis	1	0		

## Discussion

In our study, the test positivity rate for COVID-19 infection was high at 28.4%. In addition, majority of our patients had post-secondary education and were diagnosed in pregnancy during the antenatal period. The common presenting symptoms were fever, cough, malaise, sore throat, loss of taste and sense of smell. Hypertensive disease in pregnancy was the commonest diagnosed co-morbid condition and majority of the patients made satisfactory recovery from the infection. Severe COVID-19 requiring oxygen supplementation was significantly more associated with those who had coexisting morbidities at diagnosis. The World Health Organisation (WHO) advises that the test positivity rate should be less than 5% before Governments should consider the COVID-19 infection to be under control for reopening from “lockdown”. The 28.4% recorded in our result is much higher than this recommendation. It may be due toa high prevalence of COVID-19 in the community or a policy of ‘restricted access to COVID-19 testing’. At the time of our study, testing for COVID-19 was restricted only to persons with a history of travel to a place of high disease prevalence and those manifesting symptoms of the infection. A high test positivity has been suggested to indicate that the Government is only testing the sickest patients who seek medical attention and is not casting a wide enough net to know how much of the virus is spreading within its communities [12].

The clinical symptoms of fever, cough, malaise, sore throat, loss of the senses of taste and smell in our study are similar to other published reports on COVID-19 symptoms [8,13]. Furthermore, majority of our patients did not have severe disease requiring respiratory support. In a study conducted in Wuhan, China all pregnant women confirmed with COVID-19 had mild or asymptomatic disease, with fever and cough being the chief symptoms [14]. The proportion of women with severe illness requiring oxygen supplementation is similar to other studies on COVID-19 infection in pregnancy [13]. These suggest that the majority of cases of COVID-19 infections during pregnancy are asymptomatic or mild disease and may not require critical care. In this study, majority of the patients recovered satisfactorily from the infection and were discharged. Eleven were subsequently delivered either via the vaginal route or by Caesarean section for obstetric indications. This suggests that pregnant women with COVID-19 infection respond satisfactorily to standard supportive therapy and can safely deliver by the vaginal route in well selected cases. This is consistent with the findings of other studies that pregnancy did not confer additional risk for adverse outcome [10,11]. The maternal death recorded was referred from a peripheral hospital with features of puerperal septicaemia and multi-organ dysfunction 4 days after an emergency Caesarean section for obstructed labour. She died in the intensive care unit after 3 days on admission.

Majority of the newborns had normal Apgar scores and were not admitted into the neonatal unit. In addition, COVID-19 testing at 48 hours after delivery were negative for all the babies. The only perinatal death recorded in our study was a preterm delivery by emergency Caesarean section at 32 weeks in a woman referred for imminent eclampsia. The neonate, who weighed 1.2 kg died from complications of prematurity, intrauterine growth restriction and respiratory distress syndrome within 24 hours of delivery. Similar studies on foetal outcome among those delivered while diagnosed with COVID-19 infection reported that the disease did not appear to worsen perinatal outcome [15,16]. Although early perinatal COVID-19 infection suggestive of vertical transmission has been described, this was not foundin our study [15,16].

There was a significant association between COVID-19 positive women who developed severe disease requiring respiratory support either by oxygen supplementation or mechanical ventilation and the presence of hypertensive diseases of pregnancy (pre-eclampsia), a co-morbidity. Consistent with our report, Liu *et al*. [17] reported that adverse pregnancy outcomes was high, especially among those with other co-morbidities such as pre-eclampsia or other complications because respiratory syndromes may aggravate pulmonary oedema and decrease oxygen saturation. The endothelial dysfunction associated with pre-eclampsia predisposes patients to respiratory failure from pulmonary oedema. Also, COVID-19 is primarily a respiratory infection that affects the lungs. The coexistence of the two conditions may have resulted in the rapid deterioration of respiration function among the women. Our study has some limitations. The sample size is comparatively small and we did not compare our variables with those of pregnant women who did not have COVID-19. In addition, we used only negative nasal or throat swab RT-PCR test in the neonates to exclude vertical transmission. The histopathological evaluation of the placenta may have provided relevant additional information. We consider our results remain valid despite these limitations because the disease is novel with limited published scientific information on various aspects of the infection, especially from sub-Saharan Africa. There is need for further adequately powered studies to evaluate the relationship between COVID-19 in pregnancy and neonatal outcomes.

## Conclusion

In conclusion, the common presenting clinical symptoms of COVID-19 infection in pregnancy are similar to what has been reported in the non-pregnant population and majority had mild disease. The infection did not appear to worsen the maternal or perinatal outcome of pregnancy. Pre-eclampsia is a common comorbidity associated with COVID-19 in pregnancy and it appears to increase the need for respiratory support. This information is useful for decision making at the point of care when managing women with COVID-19 in infection in pregnancy.

### What is known about this topic

- COVID-19 infection can affect pregnant women;
- Its effect on pregnancy and its outcome compared to non-pregnant women is still unclear.

### What this study adds

- COVID-19 infection during pregnancy in a cohort of black African women produces symptoms similar to those seen in non-pregnant adults;
- The occurrence of pre-eclampsia with COVID-19 infection during pregnancy is significantly associated with severe disease requiring respiratory support.
